# A novel C3d-containing oligomeric vaccine provides insight into the viability of testing human C3d-based vaccines in mice

**DOI:** 10.1016/j.imbio.2017.10.002

**Published:** 2018-01

**Authors:** Yong-Gang He, Isabel Y. Pappworth, Andreas Rossbach, Joshua Paulin, Tarirai Mavimba, Christine Hayes, Liudmila Kulik, V.Michael Holers, Andrew M. Knight, Kevin J. Marchbank

**Affiliations:** a Institute of Cellular Medicine, Faculty of Medical Sciences, Newcastle University, Newcastle-upon-Tyne, NE2 4HH, UK; b School of Biomedical Sciences, Faculty of Medical Sciences, Newcastle University, Newcastle-upon-Tyne, NE2 4HH, UK; c Departments of Medicine and Immunology, University of Colorado, SOM, Denver, CO, USA; d Cardiff University, Heath Park, Cardiff, Wales, UK

**Keywords:** BM, bone marrow, CR, complement receptor, FDC, follicular dendritic cell, FO, follicular, GC, germinal centre, IC, immune complex, MZ, marginal zone, SCR, short consensus repeat, SLE, systemic lupus erythematosus, SRBC, sheep red blood cell, RT-PCR, reverse transcriptase polymerase chain reaction, Adjuvant, B cell, C3d, C4BP, Complement receptor

## Abstract

The use of C3d, the final degradation product of complement protein C3, as a “natural” adjuvant has been widely examined since the initial documentation of its immunogenicity-enhancing properties as a consequence of binding to complement receptor 2. Subsequently it was demonstrated that these effects are most evident when oligomeric, rather than when monomeric forms of C3d, are linked to various test protein antigens. In this study, we examined the feasibility of enhancing the adjuvant properties of human C3d further by utilizing C4b-binding protein (C4BP) to provide an oligomeric arrayed scaffold fused to the model antigen, tetanus toxin C fragment (TTCF).

High molecular weight, C3d-containing oligomeric vaccines were successfully expressed, purified from mammalian cells and used to immunize groups of mice. Surprisingly, anti-TTCF antibody responses measured in these mice were poor. Subsequently we established by *in vitro* and *in vivo* analysis that, in the presence of mouse C3, human C3d does not interact with either mouse or even human complement receptor 2.

These data confirm the requirement to develop murine versions of C3d based adjuvant compounds to test in mice or that mice would need to be developed that express both human C3 and human CR2 to allow the testing of human C3d based adjuvants in mouse in any capacity.

## 1 Introduction

Whilst it has been established for 40 years that complement (C) plays an important role in both initiating and shaping adaptive immunity (Pepys, 1972, Pepys, 1974), it was not until the identification and characterization of B cell complement receptor 1 (CR1; CD35) and 2 (CR2; CD21) expression [reviewed in (Cooper et al., 1988, Hourcade et al., 1989)], that the molecular basis for this was fully understood (Fearon and Carter, 1995, Hebell et al., 1991). CR1 and CR2 are encoded separately from two linked genes in man, whilst in the mouse both receptors are encoded from a single locus, *Cr2,* as a result of alternate splicing (Holers et al., 1992, Kurtz et al., 1990). On B cells, CR1 contributes to the degradation of C3b to iC3b to C3c and C3d,g in co-operation with factor I (Hourcade et al., 1989). C3d,g remains surface-bound, where further proteolytic cleavage results in the generation of the C3d fragment: both forms are ligands for CR2 (Law and Dodds, 1997), also found on the B cell plasma membrane. The generation of *Cr2* gene knockout mice (along with other key C component knockout animals) confirmed the essential roles that C and CRs play in adaptive immune responses (Ahearn et al., 1996, Molina et al., 1996).

It has been clear for some time that many components of the innate immune system act to activate and enhance the adaptive immune response, and can therefore be considered to be “natural” adjuvants (Morgan et al., 2005). C3d is one such component. This was first documented when multiple copies of mouse (m)C3d, in a linear trimer, were attached to a soluble antigen, and shown to enhance antigen-specific responses in mice up to 10,000 fold (Dempsey et al., 1996); potentially being more potent than complete Freund’s adjuvant. Unsurprisingly therefore, a number of approaches using C3 breakdown fragments as natural adjuvants have followed (reviewed by (Toapanta and Ross, 2006)). Indeed, the high degree of amino acid sequence homology between human (h) and mC3d (84.1%) suggested that hC3d-based vaccines could also be examined in mice. Furthermore studies showed that hC3 breakdown fragments induced cell cycle regulation (Melchers et al., 1985, Melchers et al., 1986) and subsequent activation (Erdei et al., 1985) of mouse B cells. In addition, polymerised hC3dg (tetramers to 20mers) was shown to be a potent activator of human B cells (Carter and Fearon, 1989) and bound readily to recombinant hCR2 or mCR2 expressed by K562 cells, implying a conserved binding site and functional homology across species with respect to C3dg binding (Martin et al., 1991, Molina et al., 1991). However, not all studies using the mC3d linear trimer have demonstrated enhanced antigen-specific immune responses (Lee et al., 2005; Suradhat et al., 2001). In addition, the mode of action of the C3d trimer is still not clear, with adjuvant properties being detected in mice in the absence of mCR2, indicating that novel receptors/interactions may exist (Lee et al., 2005, Haas et al., 2004, De Groot et al., 2014). Thus, the potential for the C3d linear trimer as a natural adjuvant for humans has been undermined significantly. However, many of these unexpected findings with this somewhat artificial form of C3d may reflect the synthetic nature of the linear trimer format, rather than a true reflection of the natural adjuvant effect of C3d/g that physiologically exists as an oligomeric “coat” on antigen surfaces.

We therefore decided to investigate whether multiple copies of C3d arrayed in an oligomeric format (using the natural C4BP oligomerisation domain), rather than linked as a linear chain, would function as an improved adjuvant. Herein, we provide new evidence that hC3d, even when arrayed with C4BP, does not enhance antigen-specific immune responses as surprisingly hC3d does not bind to mCR2 or even to hCR2 in the presence of mC3(/d). This finding clearly demonstrates the difficulty in testing hC3d-based adjuvants in the mouse. Alternative approaches to combat this include the development of wholly murine versions of such constructs or the development of mice with a fully humanised complement system (hC3 and hCR2), or molecular adjuvants designed to activate complement appropriately in multiple species.

## 2 Materials and methods

### 2.1 Cells

The Chinese hamster ovary (CHO) cell line and human B cell line Raji (expressing high levels of CR2 (Lambris et al., 1981)), were obtained from Sigma, UK (products 85050302 and 85011429, respectively). The CD19^+ve^, CR2^−ve^, mouse B cell line CH27, and transfectants expressing mCR2 (CH27.mCR2), a tetanus toxin C fragment (TTCF)-specific BCR (CH27.TTCF-BCR) or mCR2 and a TTCF-specific BCR (CH27.mCR2/TTCF-BCR), previously described (Barrault and Knight, 2004), were maintained in the presence of 0.75 mg/ml G418+/− 300 ng/ml purinomycin, 0.3 mg/ml hygromycin B.

### 2.2 Generation and expression of recombinant vaccines and controls

Plasmid pDR2ΔEF1α (Charreau et al., 1994), containing the hC4BPα chain, short consensus repeats (SCRs) 5–8 and the oligomerization domain (P04003; amino acids (aa)248-548 of the mature protein), was obtained from C. Harris (Newcastle University, UK). Using standard PCR-based cloning techniques DNA encoding the CD33 signal peptide (MPLLLLLPLLWAGALAMD) was cloned into this vector with a short linker containing restriction enzyme sites flanking a (his)tidine tag to allow for further cloning and in-frame fusion with C4BP to generate p*hC4BP*. Next, hC3d (P01024; aa 1001-1303 of pre-pro C3; with the 9th cysteine replaced by serine to remove a free sulfhydryl) was PCR cloned and incorporated in-frame between the CD33 and histag regions, generating p*hC3d-hC4BP*. hCR2 (SCR1-4)-Fc, native hC3d (containing 9th cysteine)-Fc, hC3dg (aa 955-1303) −Fc as well as Cys to Ser variants of these, mCR2 (SCR1-4)-Fc and mC3d-Fc constructs were also generated using PCR cloning utilizing a pDR2EF1α plasmid containing human IgG_1_ Fc (gift of B. Spiller, Cardiff University, UK). Recombinant TTCF encoding 444 amino acids of the mature TTCF from the 7th amino acid (Hewitt et al., 1997) was PCR cloned from the prokaryotic expression vector pET-16b with removal of the stop codon to allow read through into hC4BP sequence generating p*hC3d-TTCF-hC4BP*. Finally, p*TTCF-hC4BP* was created by PCR cloning and insertion of the TTCF product 3′ of the histag in p*hC4BP*. All plasmids were Sanger sequenced and transfected into CHO cells using the JetPEI reagent according to the manufacturer’s (Polyplus transfection) instructions. Inclusion of hygromycin B (600 μg/ml) and limiting dilution allowed the selection of clonal transfectants. Recombinant proteins were purified from tissue culture supernatant using immobilized metal ion affinity chromatography, essentially as described for TTCF (Hewitt et al., 1997), except 5 mM imidazole was used in the binding buffer.

### 2.3 Mice

All mice used in this study are on the C57BL/6 (B6) genetic background with or without the transgenes and/or genetic deletions described below.

hCR1^tg^.hCR2^tg^.*Cr2*^−/−^ mice (Pappworth et al., 2012) and hCR2^tg^.C3^−/−^ mice (Twohig et al., 2007) were routinely confirmed by PCR and immunoblot. All mice used were age and sex matched littermates from colonies maintained at Newcastle University or purchased (B6) from Harlan (UK). All experiments were performed under the terms of Animals (Scientific Procedures) Act 1986 and were authorized by the Secretary of State, Home Office, UK.

### 2.4 Preparation and immunization of protein vaccines

Animals were immunized with molar equivalent doses of antigen/construct standardized on 5 μg TTCF in sterilized PBS buffer. TTCF was also precipitated on alum according to (Ghosh et al., 1996; Adkins et al., 2000). The pellet was resuspended in PBS to achieve a final concentration 500 μg/ml alum-TTCF. Animals were immunized by intra-peritoneal (IP) injection and boosted with identical dose/antigen/route at day 28. Blood was collected prior to immunization (day 0) and then weekly until day 42.

### 2.5 Antibodies

Purified, biotin and allophycocyanin (APC) conjugated mAb 171 (mouse anti-hCR2, IgG_1_ isotype) (Guthridge et al., 2001a, Guthridge et al., 2001b); biotin mAb 7E9 (rat, anti-mCR2, IgG_2a_) (Kinoshita et al., 1988), biotin and purified 10G5 (mouse, IgG_1_ anti-TTCF, (Antoniou and Watts, 2002)) and mouse IgG_1_ isotype control were produced following standard methods. 2.4G2 (rat, anti-mCD16/mCD32, “Fc Block”), fluorescein isothiocyanate (FITC) or PerCP conjugated RA3-6B2 (rat, anti-mCD45R, B220), FITC conjugated species anti-hCD19 were obtained from Pharmingen (BD, Oxford, UK). Goat, anti-mouse IgG-HRPO; goat, anti-rabbit IgG − HRPO; donkey, anti-sheep IgG-HRPO conjugated anti-IgM or IgG, streptavidin-FITC and −PE were obtained from Jackson Immunoresearch Laboratories (Stratech Scientific, UK). Rabbit, anti-hC3d and sheep, anti-hC4BP were purchased from Dako, UK and Abcam, UK respectively.

### 2.6 ELISA

To detect the presence of recombinant proteins in CHO culture supernatant (s/n) or for measurement of anti-TTCF Ig responses, flat-bottom 96 well ELISA plates (Thermo Vinyl) were coated with undiluted s/n or 0.1 μg TTCF (in carbonate coating buffer (pH 9.6)), respectively, and incubated at 4 °C overnight. All plates were washed 3 x with PBS-0.05% Tween20 (wash buffer) and with 1% BSA/0.05% Tween20 (blocking buffer) in PBS for 2 h at room temperature (RT). For the detection of bound protein, primary antibodies were diluted 1/10,000 for the polyclonals or 1/1000 for 10G5. For the detection of anti-TCCF Ig, mouse serum was diluted in blocking buffer (1/50)) and compared to blocking buffer containing mouse anti-TTCF monoclonal antibody 10G5. Plates were then incubated for 1 h at RT and then washed 3x and blocked for additional 2 h as above. Appropriate Ig-HRPO secondary antibody was incubated in blocking buffer for 1 h at RT. Plates were washed twice with wash buffer and finally two times with PBS. Freshly made 3,3,5,5′-Tetramethylbenzidine (TMB) substrate was added to each well and the reaction stopped using 10% H_2_SO_4_. OD_450_ nm was measured using SpectraMAX 190 microplate reader (Molecular Devices, USA).

### 2.7 Flow cytometry

Mouse splenocytes were isolated as described in (Birrell et al., 2005). Human peripheral blood mononuclear cells (PBMC) were isolated from healthy volunteers using Lympholyte-H Cell Separation Media (Cedarlane Labs, Canada), according to manufacturer’s instruction and in accordance with local ethical guidance.

Splenocytes (following red blood cell lysis) or cell lines were washed in PBS containing 1% fetal calm serum (flow buffer) and then re-suspended in 10 μg/ml Fc Block for 15 min on ice. Cells were subsequently washed and re-suspended in flow buffer containing primary antibody (Ab) (0.1–3 μg/ml) for 30 min on ice. Cells were then washed 3 times in flow buffer and incubated with the appropriate secondary reagent for 30 min. Cells were washed and resuspended in flow buffer containing 1% formaldehyde. Samples were analysed using a FACS Canto ™ II (BD, Oxford, UK) and FlowJo (LLC, Ashland, OR, USA) or Cyflogic (v 1.2.1, free ware).

### 2.8 Biacore

Chips were purchased from GE Healthcare and all buffers were 0.2 μm filtered prior to use. Immobilisation: standard amine coupling reactions were carried out at 30 μl/min. Ligands were prepared in 10 mM sodium acetate at a final concentration of 2 μg/ml. Immobilisation was optimised using the pH scouting wizard program using sodium acetate at pH 5.4, 5.0, 4.6, 4.2 and 3.7. The immobilisation wizard controlled the coupling reactions. Optimal reaction conditions were set up using equal volumes of N-hydroxysuccinimide (NHS; 100 mM) and 1-ethyl-3-(dimethylaminopropyl)carbodiimide (EDC; 40 mM) the ligand in the appropriate buffer, 1 M ethanolamine-HCl pH 8.5 and 50 mM NaOH. The coupling was automatically terminated when the response unit (RU) target was reached. Typically, flow cell one (FC1) was left blank to act as control flow cell and only received NHS/EDC and 1 M ethanolamine-HCl pH 8.5. The dextran matrix in FC2 was activated with NHS/EDC, followed by ligand. Remaining free binding sites in the matrix were blocked with 1 M ethanolamine-HCl pH 8.5. Binding analysis was carried out immediately after coupling. Prior to loading of the samples, flow was allowed to stabilise for one minute. Analyte concentrations were blank buffer control (HBS-EP+; GE Healthcare, UK), 0.195 nM, 0.39 nM, 0.78 nM, 1.56 nM, 3.125 nM, 6.25 nM 12.5 nM, 25 nM, 50 nM. Analytes were injected in triplicate for 360 s. The injection was changed from sample to buffer and dissociation was monitored for 240 s. Typically the flow path was FC1 followed by FC2, which allowed subtraction of any secondary interactions with the dextran matrix. Chip surfaces were regenerated with two brief pulses of 0.5 M NaCl for 30 s.

Results were analysed using the BIAevaluation Software version 4.1. Single flow cells were analysed following blank flow cell background subtraction (e.g. FC2-FC1). Curves were aligned by y-axis transformation and values obtained for buffer controls were subtracted from the curve to compensate for buffer-surface interactions. Sensorgram data was analysed with a steady state affinity model. Good fits were indicated by low residuals and χ^2^ values (<5) as calculated by the evaluation software.

## 3 Results

### 3.1 Design and production of recombinant hC3d − TTCF multimeric vaccines

**Fig. 1:**
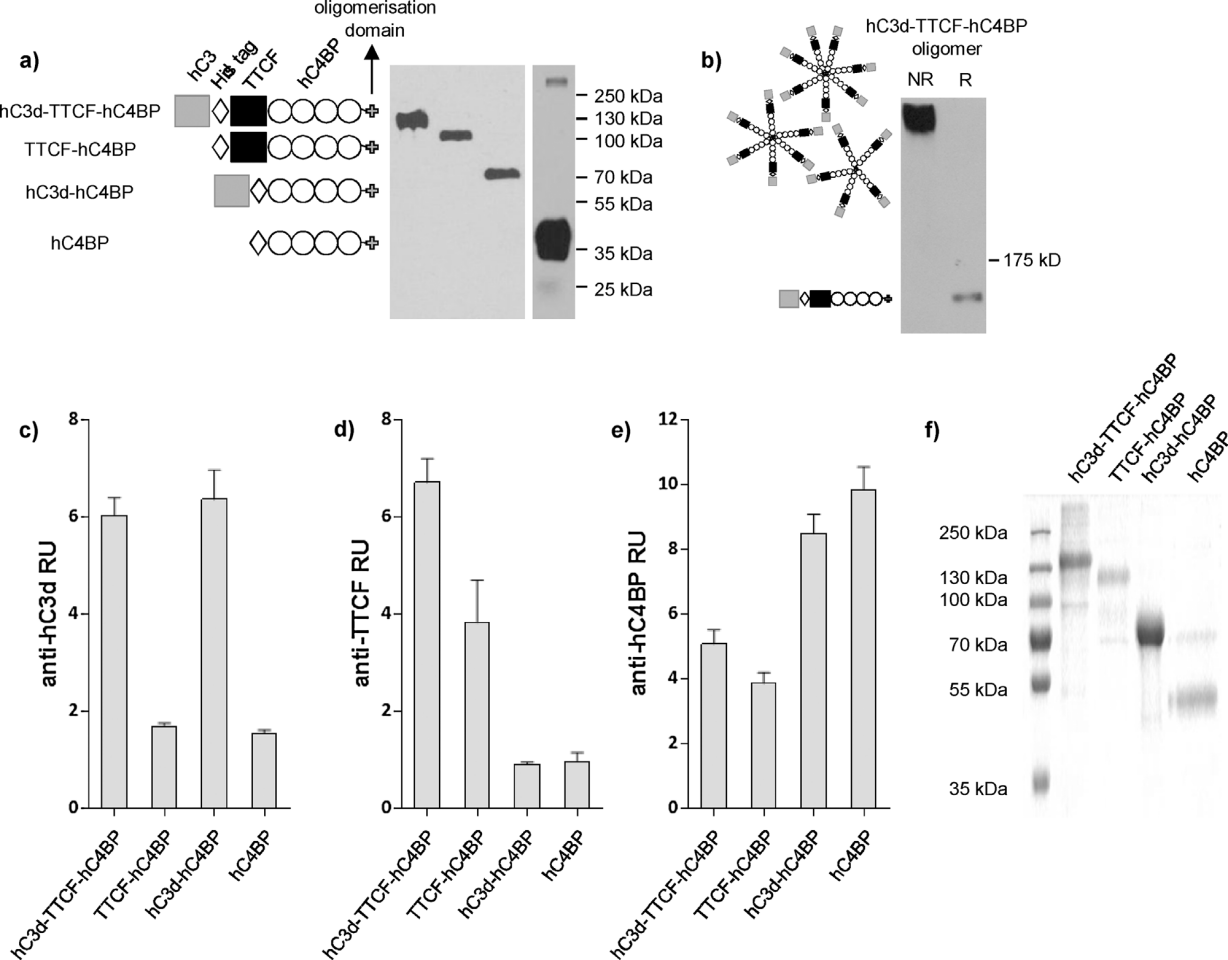
Recombinant constructs and protein production. a) Tissue culture supernatant collected from CHO cells expressing constructs, diagrammatically indicated to left of blot, was prepared in reducing sample buffer and loaded on a 10% SDS-PAGE gel before blotting to nitrocellulose. Predicted monomer constructs: hC3d-TTCF-hC4BP: 124.2 kDa, TTCF-hC4BP: 89.5 kDa, hC3d-hC4BP: 72.1 kDa and hC4BP: 38.1 kDa were detected. (b) hC3d-TTCF-hC4BP supernatant in non-reducing (NR) and reducing (R) sample buffer was separated on 5% SDS-PAGE, transferred using a modified glycine-methanol transfer buffer containing 0.1% SDS at 35 V overnight followed by 100 V for 1hr. In both panels, proteins were detected using sheep anti-hC4BP antibody followed by donkey anti-sheep IgG-HRPO. The migration of protein size markers are indicated on the right of each blot. Predicted conformation is illustrated diagrammatically either side of blots. ELISA plate wells were coated with undiluted tissue culture supernatants from CHO cell transfectants (as indicated on x axis). Plate-bound constructs were detected with rabbit anti-hC3d followed by goat anti-rabbit HRPO (c), anti-TTCF (mAb 10G5) followed by goat anti-mouse HRPO (d), sheep anti-hC4BP followed by donkey anti-sheep HRPO (e). Supernatants collected from untransfected CHO cells were used to calculate relative units (RU). Panel (f) shows coomassie blue staining following SDS-PAGE analysis of approximately 1–2ug of affinity purified recombinant proteins. All results shown are representative of several similar experiments.

Taking into account previous oligomerisation approaches (Libyh et al., 1997; Christiansen et al., 2000), we used PCR to join cDNAs such that an N-terminal hC3d was fused to the well characterised model antigen tetanus toxin C fragment (TTCF), followed by SCRs 5–8 (to act as spacers) and the C-terminal oligomerisation domain from hC4BPα chain (hC3d-TTCF-hC4BP). Various hC4BPα chain-containing control proteins were also generated (Fig. 1a). The presence of the various fusion proteins in the supernatants of CHO transfectants was confirmed using Western blotting following reducing (Fig. 1a, b) and non-reducing SDS PAGE (Fig. 1b). Using ELISA, we also confirmed both the presence and integrity of the individual domains in each fusion protein (Fig. 1c–e). Collected supernatants containing each fusion protein were subjected to immobilized metal ion affinity chromatography and eluates assessed using SDS-PAGE (Fig. 1f).

### 3.2 hC3d-TTCF-hC4BP binds to RAJI and primary human B cells through interaction with hCR2

**Fig. 2:**
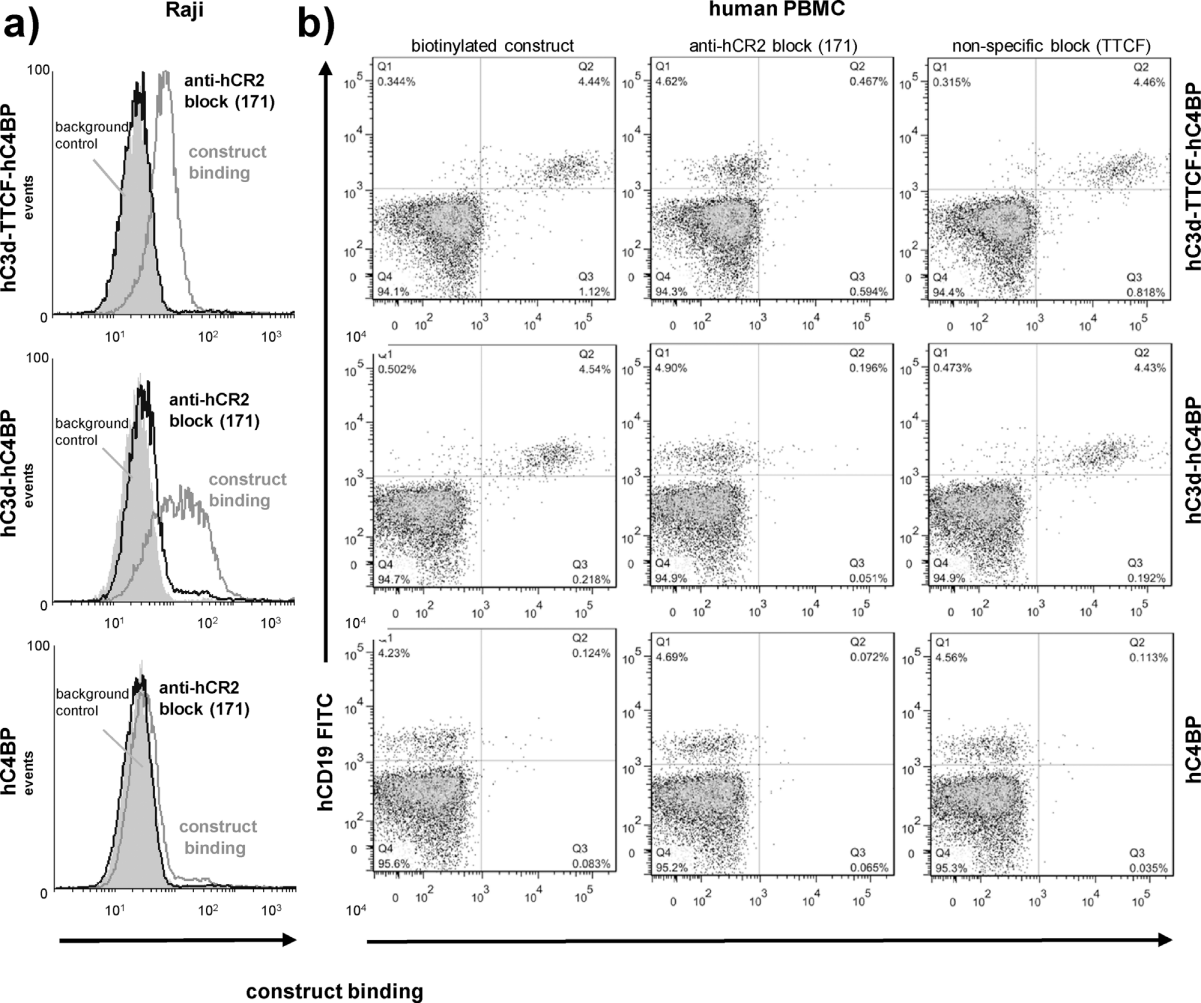
hC3d-containing constructs bind to B cells via hCR2. a) Raji cells were incubated with mAb 171 (hCR2 block, black line) or buffer alone (dark grey line) followed by hC3d-TTCF-hC4BP, hC3d-hC4BP and hC4BP. Bound constructs were detected with sheep anti-hC4BP followed by PE conjugated donkey anti-sheep IgG. The filled light grey histogram indicates the level of background staining from the sheep anti-C4BP plus secondary on cells treated with mAb 171 (but no construct) indicating no cross-reactivity of the secondary with the block (b). Isolated human PBMC cells from healthy volunteers (n = 4) were stained with anti-hCD19-FITC to label B cells and were incubated with PBS (left column), mAb 171 (middle column), or TTCF (right column) prior to incubation with biotinylated hC3d-TTCF-hC4BP, hC3d-hC4BP and hC4BP followed by SA-APC. 10,000 events were collected and results are representative of several experiments.

Next, we confirmed that the hC3d-TTCF-hC4BP candidate vaccine would specifically bind to hCR2 expressed on B cells. hCR2^+ve^ Raji B cells were incubated with the various fusion proteins in the presence or absence of an hCR2 blocking antibody (mAb 171, (Guthridge et al., 2001a, Guthridge et al., 2001b)). Both hC3d-TTCF-hC4BP and hC3d-hC4BP (but not hC4BP) fusion proteins bound specifically to hCR2 (Fig. 2a). Using CD19 to sort B cells from hPBMC, we also found that specific binding of the various biotinylated hC4BP constructs to B cell CR2 was also dependent on the presence of hC3d (Fig. 2b).

### 3.3 hC3d-TTCF-hC4BP does not induce significant anti-TTCF antibody production in mice

**Fig. 3:**
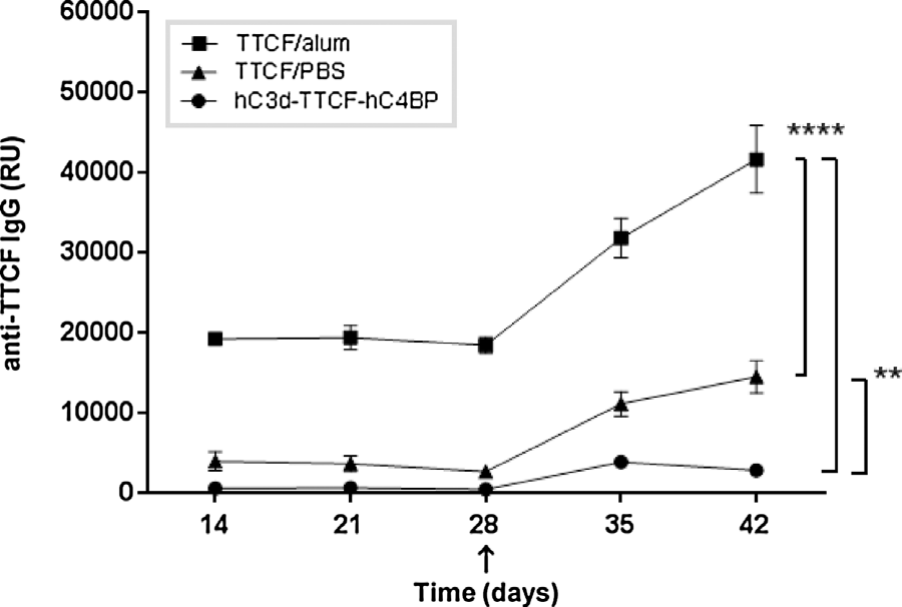
Direct *in vivo* comparison of the adjuvant effects of hC3d with the conventional adjuvant alum. Three groups (n = 4) of mice were immunized intra-peritoneally with hC3d-TTCF-hC4BP/saline (circles); TTCF/Alum (squares) or TTCF/saline (triangles) containing TTCF molar equivalents of 5 μg/animal, and boosted at day 28 (arrow) following initial immunization. Serum was collected on the days indicated and analysed for the presence of anti-TTCF IgG by ELISA. Average values (relative units (RU)) ± SD are shown. One-way ANOVA Tukey’s HSD test was used to define significance levels at 95% confidence (* p < 0.05, ** 0.01 and *** 0.001).

**Fig. 4:**
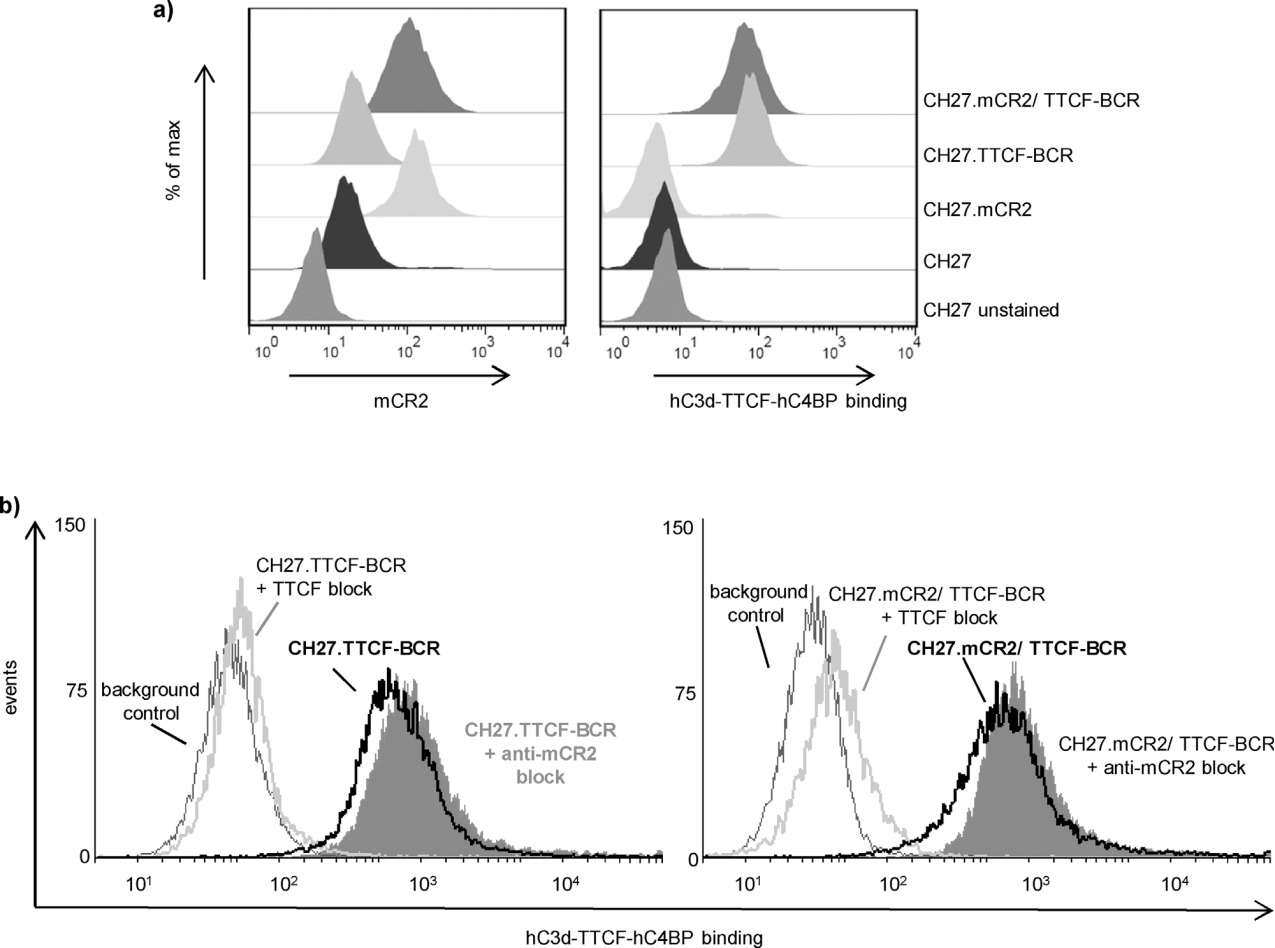
Recombinant hC3d-TTCF-hC4BP binds CH27 via TTCF specific BCR instead of mCR2. a) Untransfected CH27B cells and various CH27 transfectant clones were incubated with biotinylated anti-mCR2 monoclonal antibody 7E9 (left panel) or biotinylated hC3d-TTCF-hC4BP (right panel). Following the addition of SA-PE, fluorescence intensity was measured and histograms displayed using FlowJo. (b). Binding of biotinylated hC3d-TTCF-hC4BP to CH27.TTCR-BCR and CH27.mCR2/TTCF-BCR clones in the presence or absence of TTCF and the mCR2 blocking antibody 7G6. Binding was detected with SA-PE and histograms were overlapped using FlowJo. 10,000 cells were collected.

**Fig. 5:**
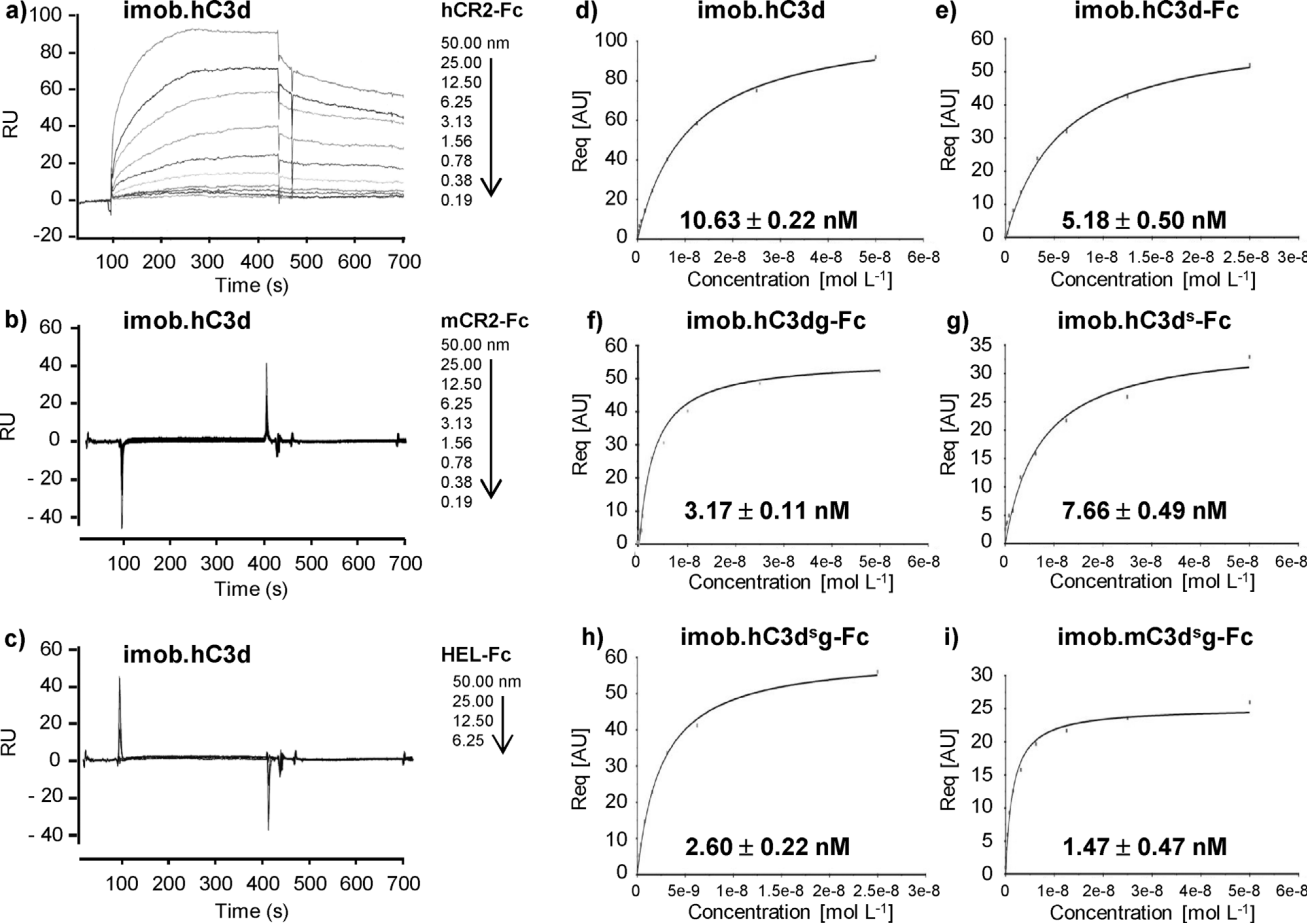
hCR2 (SCR 1–4)-Fc construct but not mCR2-Fc binds readily to immobilized serum-derived hC3d. Surface plasmon resonance sensorgrams demonstrating the association and dissociation of hCR2 SCR1-4–Fc (a), mCR2 SCR1-4 − Fc (b), and HEL − Fc (c) to immobilized monomeric native human C3d (imob.hC3d). Nanomolar concentrations of injected analyte are indicated at the right hand side of each sensorgram. Lines indicated are the normalized experimental sensorgram kinetic data. Steady state affinity graphs (d–i**)** were generated by plotting the concentrations of analyte flowed over the surface (coated with immobilized recombinant native hC3d or hC3dg Fc fusions or Cys to Ser (donated by s) variants of those molecules, as indicated, at between 94.3–96.2 RU of ligand) against the equilibrium response R_eq_ (in absorbance units, AU) of hCR2-Fc. The slope of each curve indicates higher or lower affinity of the analyte to the ligand. Calculated K_D_ values are indicated in graphs. c^2^ values showed that the curve fit described the data well (see Supplemental Table 1). Data shown is representative of triplicates.

Having established both antibody and receptor binding for the various domains in the hC3d-TTCF-hC4BP fusion protein, and following several previous reports that document hC3d interaction with mouse (m) CR1/2 (Melchers et al., 1985, Melchers et al., 1986, Erdei et al., 1985, Molina et al., 1991), we next tested the ability of hC3d-TTCF-hC4BP to act as an effective vaccine in mice. Mice were immunized with hC3d-TTCF-hC4BP, alongside others immunized with molar equivalents of TTCF precipitated with the conventional adjuvant alum, or with TTCF prepared in saline only. Following initial immunizations, all mice were boosted at day 28 (using identical antigens to the primary immunization), and then sera were collected weekly up to 42 days. Fig. 3 shows the levels of anti-TTCF IgG present in the sera collected from the various groups of mice. Somewhat surprisingly, immunization with the hC3d-TTCF-C4BP fusion protein resulted in the detection of very low levels of anti-TTCF IgG. Levels were comparable to those detected in the serum of mice immunized with TTCF in saline during the primary response but mice receiving hC3d-TTCF-C4BP fusion protein showed even lower levels of TTCF-specific IgG than those animals that received TTCF in saline following the day 28 boost. Anti-TTCF IgG levels in those mice immunized with alum-TTCF were significantly higher than those detected in mice immunized with either the hC3d-TTCF-C4BP fusion protein or recombinant TTCF in saline. In order to rule out the delivery route and antigen having a bearing on these results, we also carried out experiments using hC3d-hen egg lysozyme (HEL)-C4BP fusions via intradermal and intramuscular DNA as well as subcutaneous protein injections. Again, the immune response to the hC3d – Antigen-C4BP vaccines was poor (suppl. data Fig. 1, Fig. 2, Fig. 3, Fig. 4, Fig. 5, Suppl. Table 1).

### 3.4 hC3d-TTCF-C4BP does not bind to mCR2

Our finding that mice immunized with hC3d-TTCF-C4BP showed very low levels of TTCF-specific IgG, led us to re-evaluate the reported interactions of hC3d with mCR2 (Melchers et al., 1985, Melchers et al., 1986, Erdei et al., 1985, Molina et al., 1991). For this, we firstly utilized previously generated transfectants of the mCR1/2 negative mouse B cell line CH27, which expressed combinations of mCR2 and a TTCF-specific, hIgG_1_ BCR (Barrault and Knight, 2004). Fig. 4 shows that biotinylated hC3d-TTCF-C4BP bound those CH27 transfectants expressing a TTCF-specific BCR (CH27.mCR2/TTCF-BCR and CH27.TTCF-BCR; Fig. 4a, right panel) and that binding to both these transfectants was inhibited by prior incubation with recombinant TTCF protein (Fig. 4b, left panel). In addition, as expected hC3d-TTCF-C4BP did not bind to untransfected CH27B cells (Fig. 4a), but nor was it seen to bind to CH27B cells expressing just mCR2 (CH27.mCR2), although mCR2 expression was confirmed by anti-mCR2 staining (Fig. 4a, left panel). This was also confirmed by the finding that hC3d-TTCF-C4BP binding to CH27.mCR2/TTCF-BCR cells was not inhibited in the presence of a blocking anti-mCR2 mAb (Fig. 4b, right panel). These finding indicate that hC3d-TTCF-C4BP binding to CH27.mCR2/TTCF-BCR cells could be solely attributed to TTCF/BCR interactions and that hC3d-TTCF-C4BP unexpectedly does not bind mCR2.

To ensure that the lack of hC3d-TTCF-C4BP binding to mCR2 was not just a feature of the recombinant C3d in the fusion protein, we next measured the binding of native hC3d purified from human serum, along with an alternative recombinant form of hC3d, to soluble mCR2 and hCR2 using surface plasmon resonance (SPR). As seen in Fig. 5, we found that a hCR2 (SCR 1-4)-Fc construct bound readily to immobilized serum-derived hC3d under physiological salt conditions with an apparent K_D_ of 10.63 ± 0.22 nM; (supplemental Table 3, Fig. 5a). This is significantly (>10 fold) higher than that noted in previous studies using monomeric C3d and recombinant monomeric hCR2 (SCR1-2, 1-4 or 1-15) either as ligand or analyte with low salt conditions (50 mM) that were deemed to provide optimal binding in this context (Asokan et al., 2006 Guthridge et al., 2001a, Guthridge et al., 2001b, Sarrias et al., 2001). In contrast to the strong binding of hCR2-Fc to immobilized hC3d, use of mCR2-Fc as the analyte showed no binding to hC3d (Fig. 5b) above that seen with an irrelevant Fc fusion protein (Fig. 5c), supporting the flow cytometry data using CH27 cells expressing mCR2 (Fig. 4). We also extended this analysis to evaluate the binding of a number of different hC3d variants (i.e hC3d, a Cys to Ser variant of hC3d(s), hC3d with the g fragment attached and a Cys to Ser hC3d,g (s) variant; all in Fc fusion form) to hCR2 − Fc. As seen in Fig. 5h, we found that hCR2-Fc bound to immobilized hC3d,g (s) with the highest affinity (2.6+/−0.22 nM), in fact, hCR2–Fc bound better to all hC3d − Fc constructs than it did to monomeric hC3d (Fig. 5d–g). Thus, whilst there were marginal differences in hC3d(g) or mutated hC3d binding to hCR2 SCR1-4 these data confirm that recombinant hC3d-TTCF-C4BP failed to bind mCR2 as did recombinant monomeric hC3d in contrast to that reported previously (Melchers et al., 1985, Melchers et al., 1986, Erdei et al., 1985, Molina et al., 1991). Interestingly, we also demonstrated that hCR2-Fc bound mC3d − Fc immobilized on the chip with an affinity of 1.47 ± 0.47 nM, which was 4 fold higher than the affinity measured for the interaction of hCR2-Fc with hC3d-Fc immobilized on the chip surface.

### 3.5 hC3d-TTCF-C4BP only binds hCR2 expressed by transgenic mouse splenic B cells isolated from C3^−/−^ animals

**Fig. 6:**
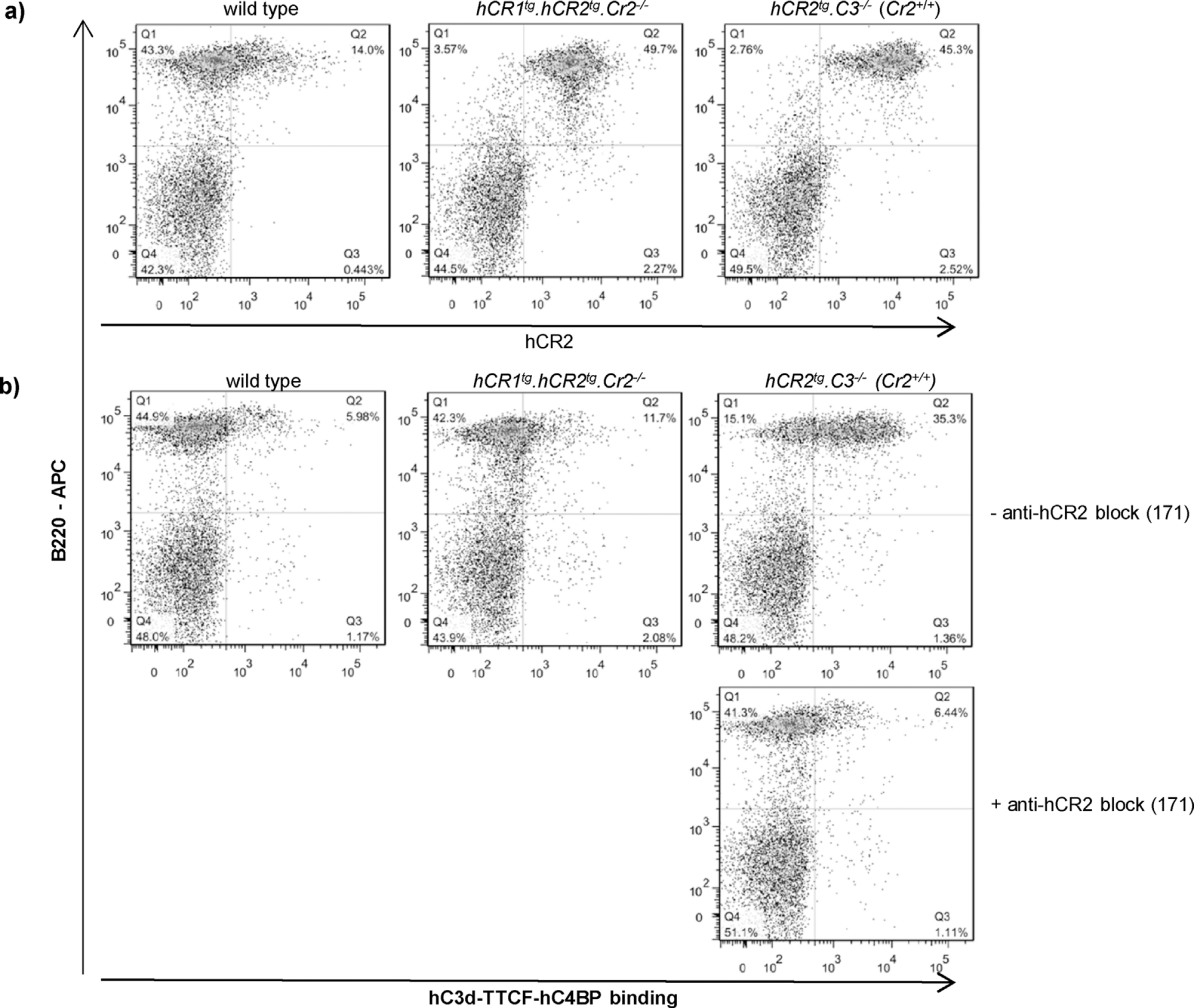
Recombinant hC3d-TTCF-hC4BP binding to hCR2 expressed on transgenic mouse B cells is influenced by the presence of mC3. Isolated splenocytes (genotypes indicated above) were stained with (a) biotinylated anti-hCR2 mAb 171 (x axis) and anti-B220-APC to identify B cells (y axis) or (b) biotinylated hC3d-TTCF-hC4BP and anti-B220-APC followed by SA-PE. In addition splenocytes from *hCR ^tg^. C3^−/−^(Cr2^+/+^)* were also stained with biotinylated hC3d-TTCF-hC4BP and anti-B220-APC followed by SA-PE, in the presence unlabeled mAb 171 (lower panel). Panels shown are representative of three separate experiments.

As a result of our demonstration that hC3d-TTCF-C4BP (along with monomeric hC3d) does not bind mCR2, we re-examined the ability of the hC3d-TTCF-C4BP candidate vaccine to enhance anti-TTCF antibody production following the immunization of transgenic mice expressing hCR2. Using an essentially identical immunization and boost protocol to that shown in Fig. 3, we immunized groups of >5 animals from two independent lines of hCR2 transgenic mice (Marchbank et al., 2002, Kulik et al., 2011) with hC3d-TTCF-C4BP, TTCF-alum and TTCF in saline. As before, the levels of TTCF-specific IgG measured in the sera of these mice (suppl. data Figs. 6, suppl. Table 4) were essentially the same as those shown previously for non-transgenic mice (Fig. 3). To explore possible reasons to explain the lack of enhanced TTCF-specific antibody in hCR2 transgenic mice, we carried out further binding analysis with *ex-vivo* B cells isolated from either B6 non-transgenic, or hCR1/2 transgenic mice. Similar to that seen with mouse B cell lines (Fig. 4), hC3d-TTCF-C4BP did not bind to B cells isolated from B6 mice (Fig. 6b). In addition, in contrast to that seen with human PBMC or human B cell lines (Fig. 2), Fig. 6b also shows that hC3d-TTCF-C4BP also fails to bind to mouse B cells isolated from hCR2 transgenic mice, despite their expression of hCR2 (Fig. 6a). To examine a possible reason for this discrepancy, and in light of the increased binding of mC3d to hCR2 in SPR analysis, we also examined the binding of hC3d-TTCF-C4BP to B cells isolated from hCR2 transgenic mice that had been crossed with mice lacking C3 (*C3^−/−^)*. Similar to that seen with both human B cell lines and PBMC (Fig. 2), Fig. 6b shows clearly that hC3d-TTCF-C4BP does specifically bind to hCR2 expressed by B cells isolated from hCR2 transgenic mice that lack C3 (hCR2/*C3^−/−^)*.

## 4 Discussion

The key role played by complement in enhancing the adaptive immune response is now well accepted. Over 40 years ago, the seminal studies by Pepys et al. using C3 activating/depleting agents including cobra venom factor and zymosan clearly demonstrated intact C3 function was important for the T-dependent response to various antigens including sheep red blood cells and ovalbumin (Pepys, 1972, Pepys, 1974). A refined molecular mechanism explaining this effect was established by Fearon and colleagues(Fearon and Carter, 1995) who then exploited this knowledge and established that multiple copies of C3d, in a linear trimer, could enhance antigen-specific responses up to 10,000 fold (Dempsey et al., 1996). However, the initial potential of trimeric C3d, as a highly potent molecular adjuvant, has not been realized and the reason(s) for this remain(s) unclear. One possible explanation is that the artificial linear trimer structure, when attached to the majority of test antigens, fails to represent naturally opsonised antigen, and consequently does not provide sufficient CR cross-linking for a functional threshold to be reached. It is therefore possible that attaching more C3d to test antigens could overcome this limitation leading to enhanced adjuvant activity. In order to test this hypothesis, in this study we have attached C3d via the natural oligomerization domain from hC4BP to a test antigen to generate C3d − Ag multimers. Our analysis of the product formed from this construct clearly demonstrates that high order multimers are successfully produced and can be purified from mammalian cell cultures. Importantly, we also demonstrate that both the test antigen and C3d components within these multimers are likely folded correctly (as expected from expression in a mammalian cell line compared with other forms of bio-production) as evidenced by the fact that the individual domains within the multimer retain specific binding to their appropriate receptors expressed on immortalized B cell lines as well as primary human B cells. Despite these properties however, the ability of two versions of this prototype vaccine (HEL and TTCF) to enhance antigen-specific immune responses in mice was found to be very poor.

Following further investigations we, somewhat surprisingly, established that the hC3d component of this construct, along with purified hC3d, does not bind mCR1/2. This finding apparently contradicts studies using polymerised human complement C3dg (pC3dg) and its specific binding to COS and K562 cells expressing recombinant mCR2 (Martin et al., 1991). These data were also apparently supported by independent experiments using erythrocytes coated with antibody and human complement (EAC3d) binding murine AJ9 B cells, albeit less well than human B cells (Fingeroth et al., 1989) and where EAC3d,g bound to the surface of K562 cells expressing recombinant mCR2 which was blocked by mAb 7G6 (Molina et al., 1991). They also reported that glutaraldehyde-cross-linked hC3d,g bound both recombinant mCR2 and hCR2 with almost identical kD of 4.6 nM (Molina et al., 1991). Our SPR analysis, with hC3dg −Fc and hCR2 confirm a similar binding affinity (kD of 3.1–5.1 nM) but in contrast we did not detect any interaction between immobilized hC3d, either as monomer or as an Fc-fusion product, with mCR2-Fc (Fig. 5). This finding is also supported by our studies using recombinant multimeric hC3d and mouse B cell lines or splenic B cells (Figs. 4 & 6).

Using purified and recombinant proteins in the SPR experiments under physiological salt conditions, along with the flow cytometric analysis of mouse B cells, we conclude with considerable confidence that the lack of binding of hC3d to mCR2 detected in these experiments represents the *in vivo* setting, and that unknown factors may have influenced the studies using recombinant mouse CR2 expressed on K562 (Martin et al., 1991, Molina et al., 1991). In the case of Martin et al., as the molecule expressed was a hybrid of hCR2 and the 2 NH-terminal SCRs of mCR2 it is possible highly polymerised hC3dg may have bound another domain of hCR2 or a neo epitope generated in the hybrid CR2 protein or the glutaraldehyde cross-linked pC3dg, in any cases both proteins were essentially synthetic constructs and not in their native conformation. This does not completely explain the findings by Molina et al., although the affinity analysis in this study was also carried out with a similar glutaraldehyde cross-linked human C3d, their initial binding studies used erythrocytes coated with human complement components and where blocked by an antibody (7G6) known to block mouse C3d interactions (Molina et al., 1991, Kinoshita et al., 1990). However, these data are in conflict with the earlier work using antibody-complement based rosette assays with mouse splenocytes or mouse B cell lines which indicated little or significantly reduced interaction between human C3d and mouse complement receptors (Dierich et al., 1974), (Fingeroth et al., 1989).

A second equally surprising finding from our study is that hC3d binding to hCR2 expressed by mouse B cells was only evident when the B cells were isolated from mice lacking C3. This strongly suggests that mC3d successfully competes with hC3d for binding to hCR2. This was supported by our SPR binding data (Fig. 5i). Thus, these findings suggest that use of transgenic mice expressing hCR2 for the testing of hC3d-based molecular adjuvants is severely limited.

The oligomerisation domain on the complement regulator C4BP (Scharfstein et al., 1978) has previously been exploited to increase valency of either recombinant antibodies (Libyh et al., 1997; Oudin et al., 2000) or a viral receptor (Christiansen et al., 2000). This led us to conceive an oligomeric vaccine structure based on C4BP that would have three functionally established benefits. Firstly, it would act as oligomeric antigen carrier, secondly, by containing hC3d, it would target B cells (via CR2) and thirdly, via the C4BP oligomerisation domain (the last 60 C-terminal amino acids of the C4BP alpha chain), it would also act as a T cell adjuvant (Forbes et al., 2012). The combination of the C3d/B cell interaction with the T cell adjuvant effects of the oligomer compound was thus predicted to enhance immunity to the TTCF test antigen significantly compared to immunizing animals with antigen alone. However, we found no evidence of any adjuvant effect when measuring anti-TTCF specific antibodies. This also suggests the oligomerisation domain of hC4BP does not provide CD4^+^ T cell help for any anti-TTCF B cells in the mouse. Furthermore, no advantage of oligo-antigen valency over monomeric antigen was realized, although this was likely compounded by the lack of interaction between hC3d and m(or) hCR2.

The full testing of these oligomeric hC3d antigen compounds in the recently generated hCR2 BAC mice (Kulik et al., 2011) crossed onto the C3^−/−^ background would be possible given time. However, these experiments will be hampered by the loss of endogenous C3 to drive and support the immune response and therefore give a true idea of how that will translate to the clinical setting that these pre-clinical studies are supposed to emulate. We concede that only construction of mC3d fused to mC4BP constructs will allow the adjuvant capabilities of similar oligomeric C3d compounds to be fully tested in mouse models. This remains an important line of investigation as there are still many unknowns regarding the mode of action of C3d as an adjuvant. The linear trimer construct of C3d has been shown to be an effective stimulator of B cell responses but monomeric C3d-Ag is considered immunosuppressive (Dempsey et al., 1996). Thus, would an arrayed C3d structure function as a group of monomers in this context or act similar to the linear trimer? Potentially, C3d-containing multimers may also result in reduced immune response due to excessive CR2 cross-linking that has been reported with the use of high doses of C3dg-streptavidin complexes (Lee et al., 2005).

In summary, these experiments clearly demonstrate that hC3d does not bind to mCR2 as previously reported, while mC3d does bind hCR2 with an increased affinity to that measured for hC3d. Importantly we also demonstrate for the first time that even following the replacement of mCR2 with the hCR2 homologue in transgenic animals no hC3/hCR2 binding is seen in the present of mC3. These 2 new findings severely hamper the testing of novel strategies to improve the efficiencies of hC3d-containing recombinant adjuvants in mice. Importantly these studies thus highlight the importance of extensive testing of candidate C3-based vaccines and that, even when attempts are made to maximize species conservation in animal models, considerable care needs to be taken when analyzing cross species receptor/ligand interactions.

## Conflict of interest

There are no conflicts of interest.
