# Supplementary material for: A novel C3d-containing oligomeric vaccine provides insight into the viability of testing human C3d-based vaccines in mice
Source: Immunobiology. 2018 Jan;223(1):125–34. doi: 10.1016/j.imbio.2017.10.002 (PMC5849677; doi:10.1016/j.imbio.2017.10.002)
Supplement: Supplementary file 1 [file mmc1.pptx]

## Slide 1
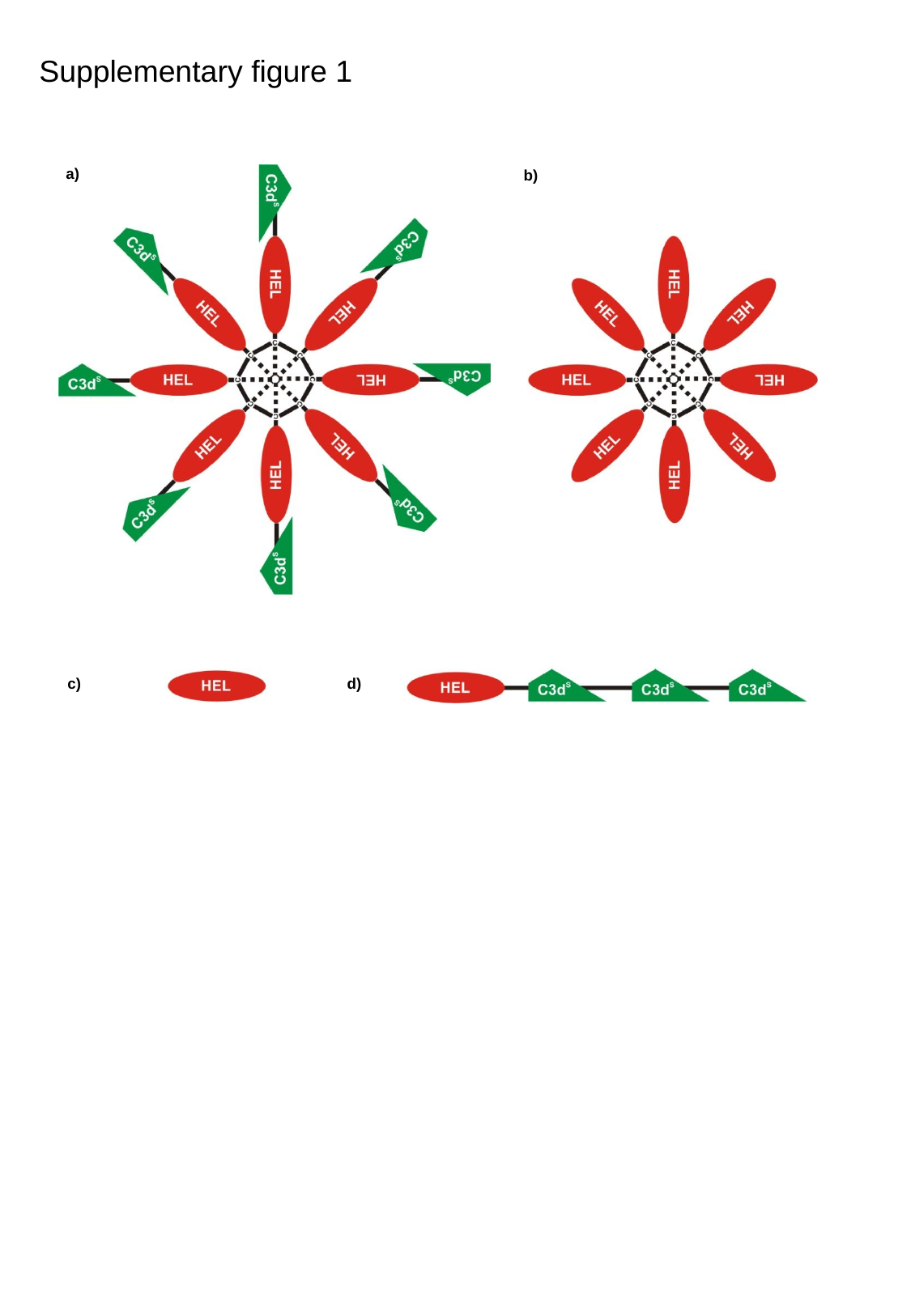

Supplementary figure 1
a)
b)
c)
d)

## Slide 2
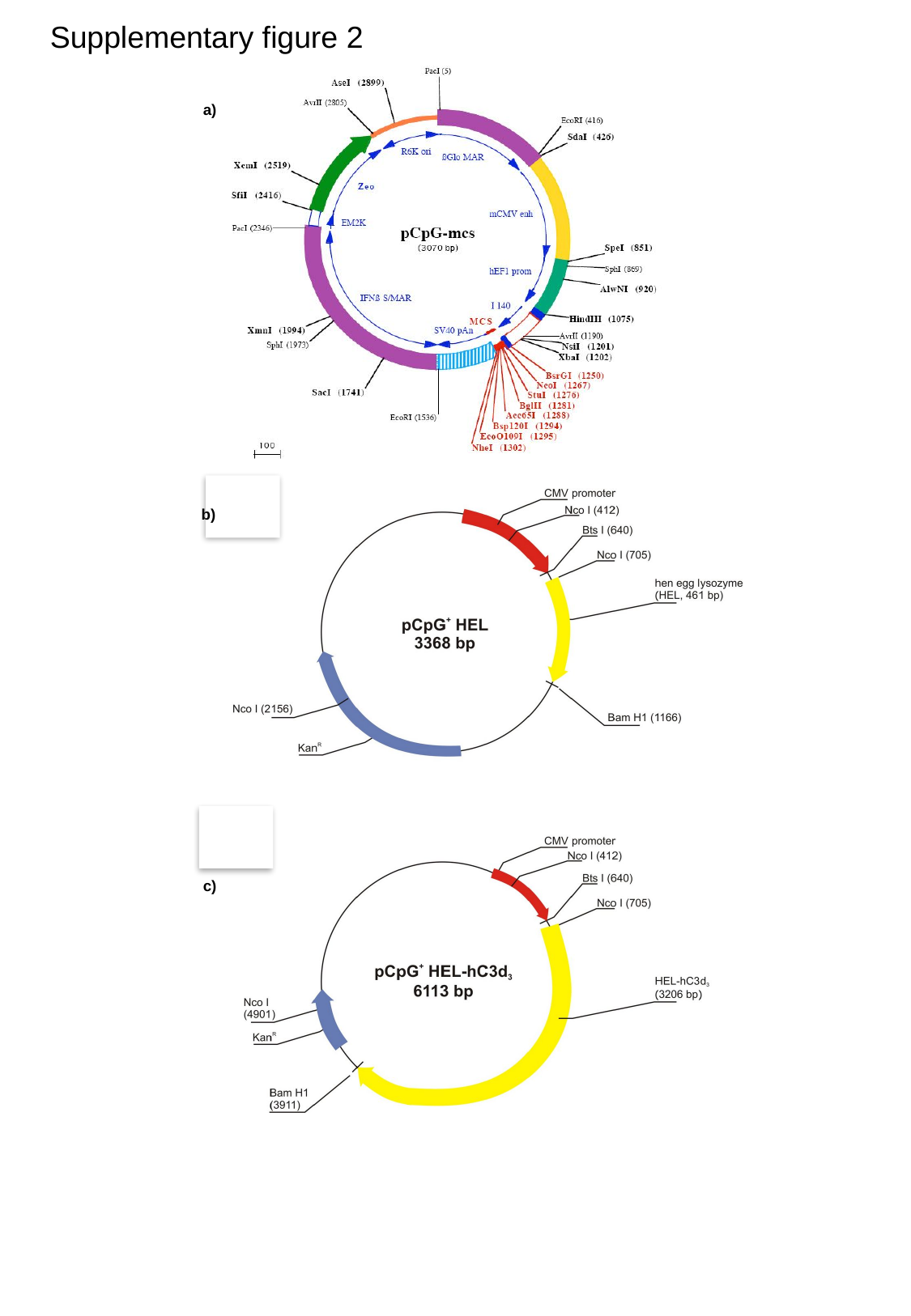

Supplementary figure 2
a)
b)
c)

## Slide 3
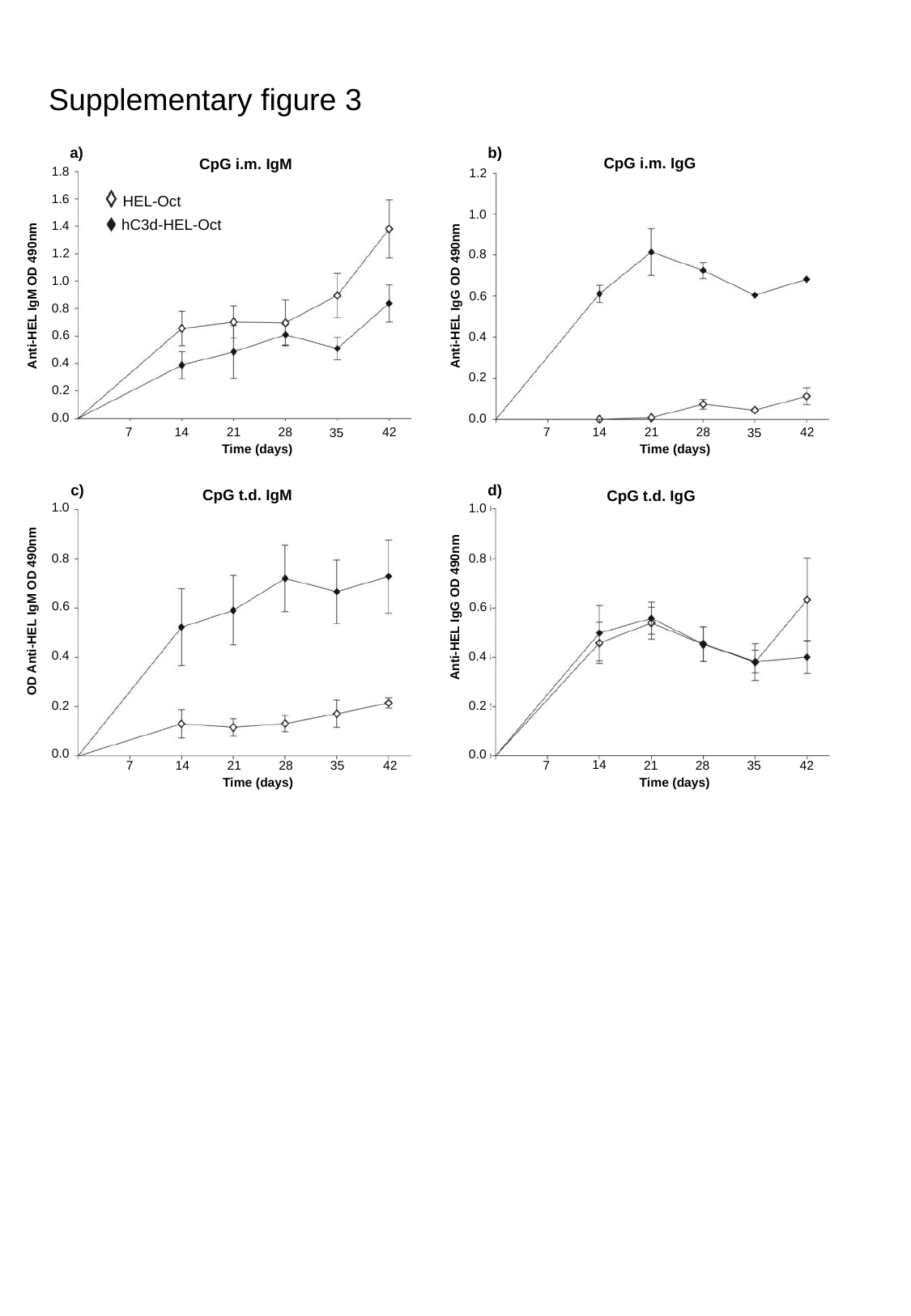

Supplementary figure 3
a)
b)
CpG i.m. IgG
CpG i.m. IgM
1.8
1.6
1.4
1.2
1.0
0.8
0.6
0.4
0.2
0.0
Anti-HEL IgM OD 490nm
1.2
1.0
0.8
0.6
0.4
0.2
0.0
Anti-HEL IgG OD 490nm
HEL-Oct
hC3d-HEL-Oct
14
7
28
21
42
35
Time (days)
14
7
28
21
42
35
Time (days)
d)
c)
CpG t.d. IgM
CpG t.d. IgG
1.0
0.8
0.6
0.4
0.2
0.0
OD Anti-HEL IgM OD 490nm
1.0
0.8
0.6
0.4
0.2
0.0
Anti-HEL IgG OD 490nm
14
7
28
21
42
35
Time (days)
14
7
28
21
42
35
Time (days)

## Slide 4
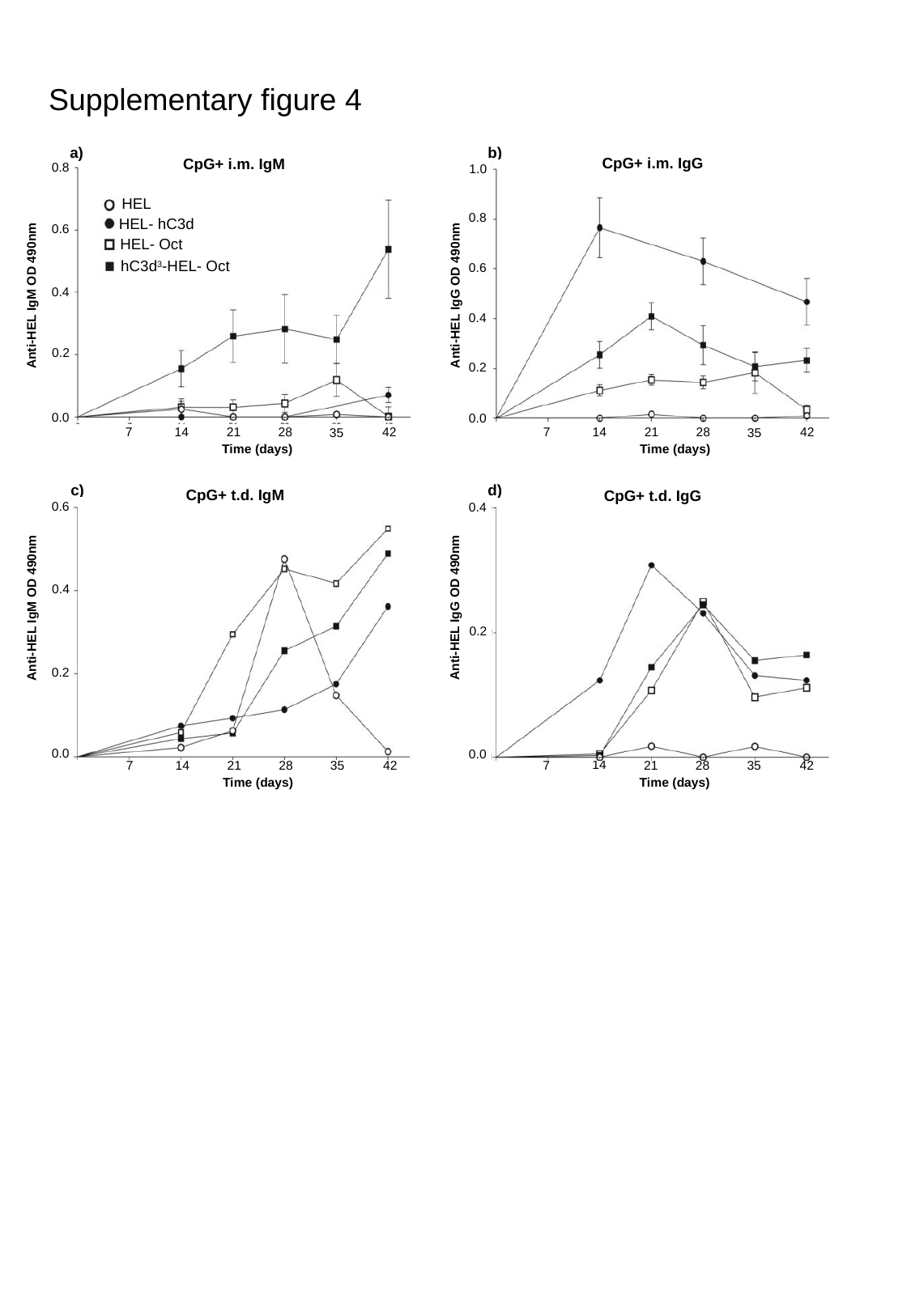

Supplementary figure 4
a)
b)
CpG+ i.m. IgG
CpG+ i.m. IgM
0.8
0.6
0.4
0.2
0.0
Anti-HEL IgM OD 490nm
1.0
0.8
0.6
0.4
0.2
0.0
Anti-HEL IgG OD 490nm
HEL
HEL- hC3d
HEL- Oct
hC3d3-HEL- Oct
14
7
28
21
42
35
Time (days)
14
7
28
21
42
35
Time (days)
d)
c)
CpG+ t.d. IgM
CpG+ t.d. IgG
0.6
0.4
0.2
0.0
Anti-HEL IgM OD 490nm
0.4
0.2
0.0
Anti-HEL IgG OD 490nm
14
7
28
21
42
35
Time (days)
14
7
28
21
42
35
Time (days)

## Slide 5
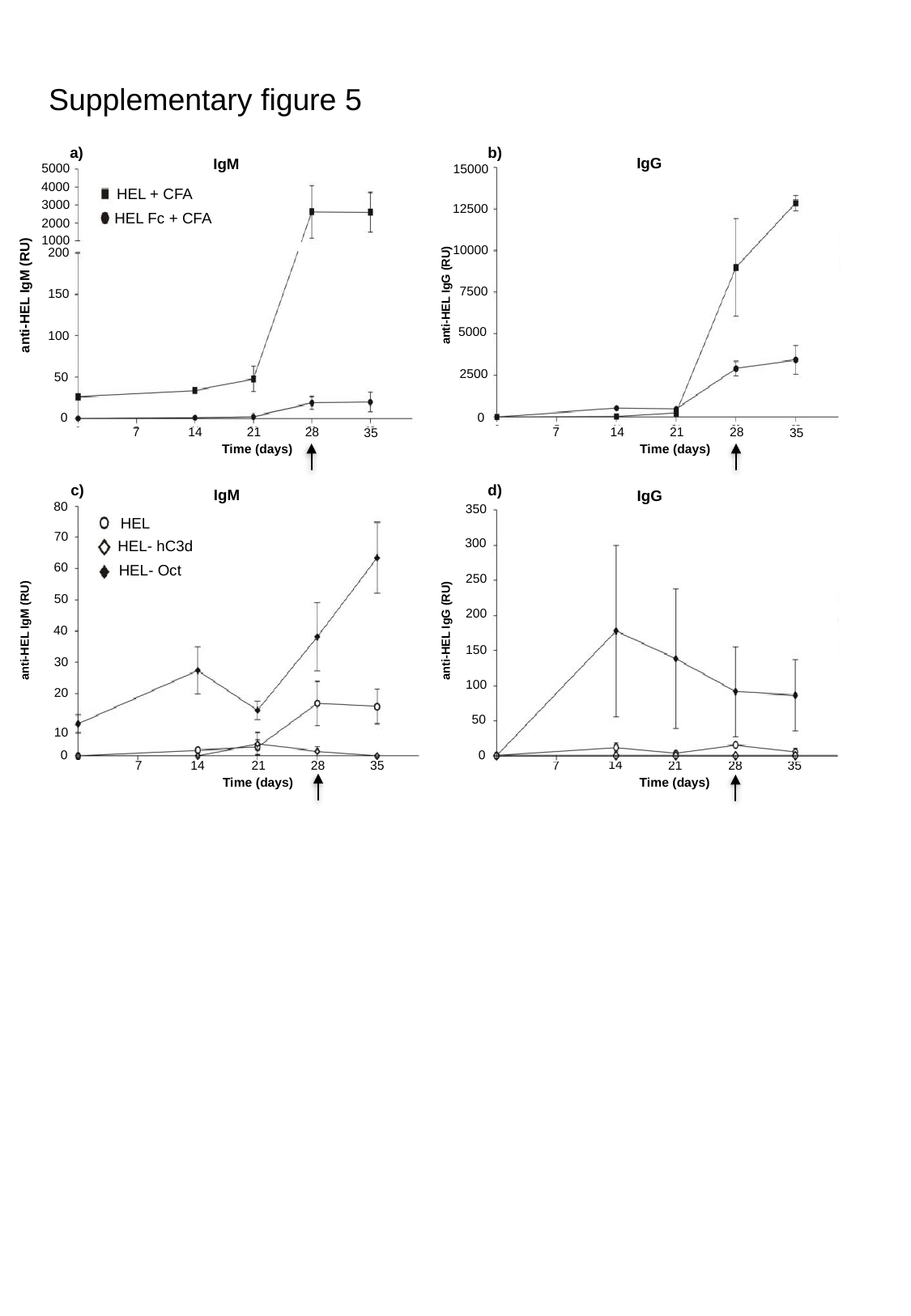

Supplementary figure 5
a)
b)
IgG
IgM
5000
15000
4000
HEL + CFA
3000
12500
HEL Fc + CFA
2000
1000
10000
200
anti-HEL IgG (RU)
7500
150
anti-HEL IgM (RU)
5000
100
2500
50
0
0
14
14
7
7
28
28
21
21
35
35
Time (days)
Time (days)
d)
c)
IgM
IgG
80
350
HEL
70
300
HEL- hC3d
60
HEL- Oct
250
50
200
anti-HEL IgG (RU)
anti-HEL IgM (RU)
40
150
30
100
20
50
10
0
0
14
7
28
14
21
7
35
28
21
35
Time (days)
Time (days)

## Slide 6
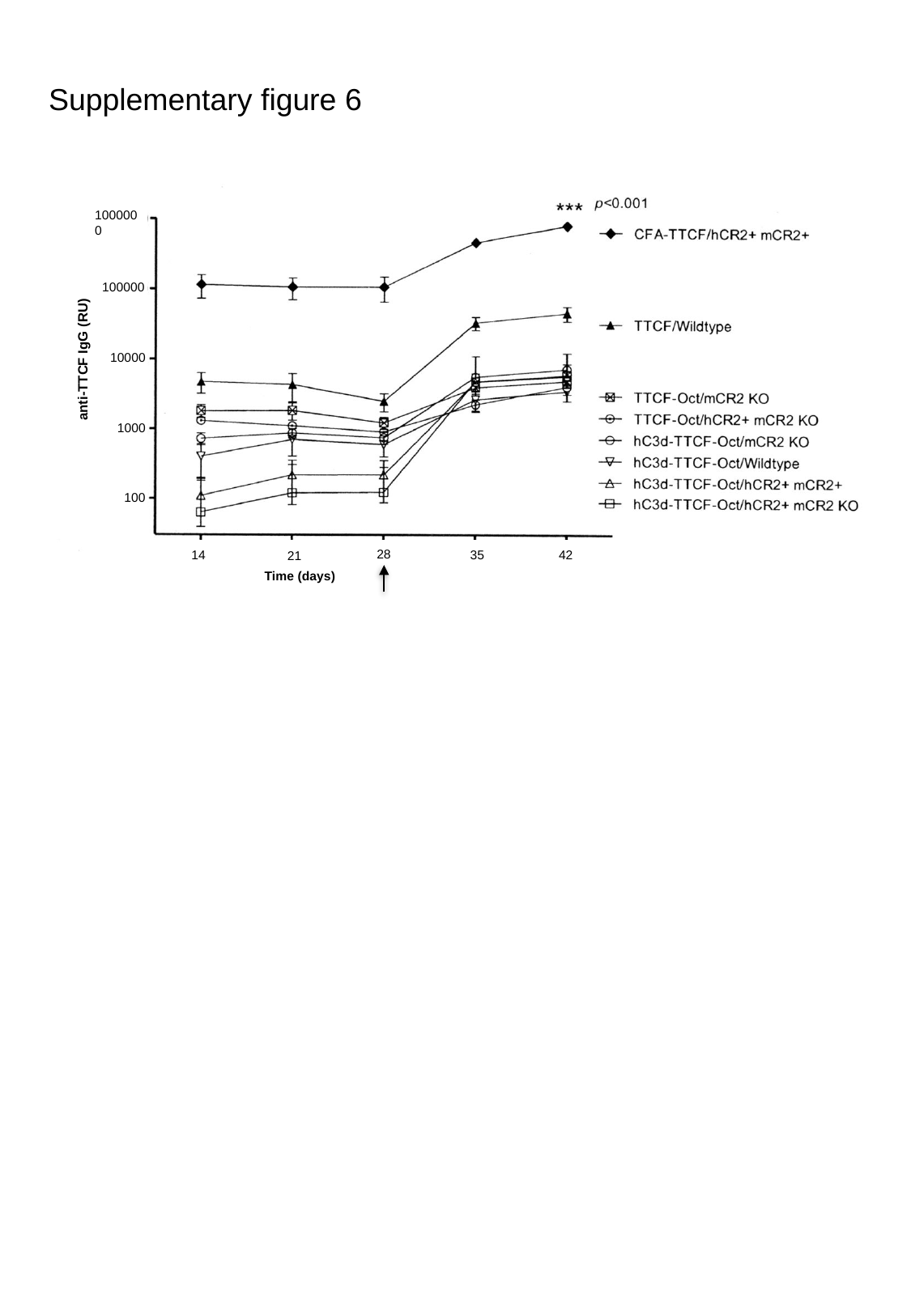

Supplementary figure 6
1000000
100000
anti-TTCF IgG (RU)
10000
1000
100
28
14
35
42
21
Time (days)
