# Supplementary material for: A novel C3d-containing oligomeric vaccine provides insight into the viability of testing human C3d-based vaccines in mice
Source: Immunobiology. 2018 Jan;223(1):125–34. doi: 10.1016/j.imbio.2017.10.002 (PMC5849677; doi:10.1016/j.imbio.2017.10.002)
Supplement: Supplementary file 2 [file mmc2.docx]

**Supplementary Figure 1: Schematic of the vaccine constructs used in this phase of the study.** Panels A through D show a diagrammatic representation of the four proteins to be expressed by the vaccine vectors. Shown are the octameric proteins (see supplementary materials and methods), one containing the human C3d^S^ adjuvant, (^S^ signifying serine swapped for cysteine used for disulphide bonding of C3d to substrate) (a) and the other without adjuvant (b). Additional DNA vaccine constructs included HEL (hen egg lysozyme) alone (c) and HEL-hC3d_3_ (d).

**Supplementary Figure 2: CpG and CpG+ vector diagrams for the administered DNA vaccines**

a) CpG^-^ containing a multiple cloning site b) CpG^+^ HEL (c) CpG^+^ HEL-hC3d^S^_3._

**Supplementary Figure 3: Ig responses to the adjuvant constructs using CpG low vectors as DNA vaccines administered by intramuscular injection or biolistic transfer**. Anti-HEL Ig titres were measured by absorbance at 490 nm (y axis) over time (x axis). C57Bl/6 WT mice received vaccine vector DNA by intramuscular injection (panels a and b) or biolistic transfer (panels c and d) at day 0. Sera were collected from three to six mice per group weekly from day 14 until day 42. IgM (panels a and c) and IgG (panels b and d) were screened against native HEL in an ELISA-based assay. Empty vector control responses have been subtracted from the traces on the graphs. Data shown is average of each group consisting of 6 mice ± standard error of the mean.

**Supplementary Figure 4: Ig responses to CpG+ (containing) DNA vaccines administered by intramuscular injection or biolistic transfer**.

Ig titres measured by absorbance at 490 nm (y axis) over time (x axis). C57Bl/6 WT mice received vaccine vector DNA by intramuscular injection (panels a and b) or biolistic transfer (panels c and d) at day 0. Sera were collected from three to six mice per group weekly from day 14 until day 42. IgM (panels a and c) and IgG (panels b and d) were screened against native HEL in an ELISA-based assay. Empty vector control responses have been subtracted from the traces on the graphs. Data shown from one experiment; each group consisted of 6 mice except t.d. CpG^+^ HEL-Oct (n = 5). Error bars are standard error of the mean but have been omitted in panels C and D for the purpose of clarity.

**Supplementary Figure 5: IgM and IgG antibody titres following subcutaneous injection of proteinaceous vaccines**.

Vaccines were injected subcutaneously. Top row: HEL + CFA and HEL Fc + CFA; (a) IgM, (b) IgG. The two bottom graphs show the antibody titres in response to HEL Fc, hC3d^S^-HEL-Oct and HEL-Oct all injected in saline solution: (c) IgM, (c) IgG. Mouse sera were collected at the indicated time points and analysed using native HEL in an immunosorbent assay. Mice were boosted with native HEL in saline at day 28 (indicated by arrow). Antibody titres are shown on the y-axis, calculated as the reciprocal of the dilution factor at absorbance at 490 nm. Data was derived from one experiment; HEL + CFA: n = 2, HEL Fc + CFA: n = 6, HEL Fc: n = 4, hC3d^S^-HEL-Oct: n = 6, HEL-Oct: n = 6; Error bars: SEM.

**Supplementary Figure 6: Direct comparison of adjuvant effects *in vivo* of hC3d in hCR2 tg and control mice**

Mouse (m) CR1/2 knockout mice were also immunised to establish if the hC3d-C4BP-TTCF octamer had adjuvant effects in the absence of hC3d interaction with

mCR2. Mice were immunised in a protocol identical to that carried out previous experiment using wild type animals with the exception that the conventional adjuvant control used in this experiment was Complete Freund's Adjuvant (CFA). Mice were immunised in Denver and Newcastle and collected sera was stored at < -20c until all samples were obtained. Denver samples were sent back to Newcastle for antibody response analysis
