# Supplementary material for: A novel C3d-containing oligomeric vaccine provides insight into the viability of testing human C3d-based vaccines in mice
Source: Immunobiology. 2018 Jan;223(1):125–34. doi: 10.1016/j.imbio.2017.10.002 (PMC5849677; doi:10.1016/j.imbio.2017.10.002)
Supplement: Supplementary file 3 [file mmc3.docx]

## Supplementary Materials and Methods

###

### Experimental Animals

*Experiments with Hen egg lysozyme*

Harlan (Surrey, UK) supplied 7 – 9 week old male C57Bl/6 mice, which were housed in conventional cages under the care of the staff at Biomedical Services (BMS) experimental animal facility at Cardiff University. Mice were housed in specific pathogen-free conditions and handled according to Home Office guidelines.

*Experiments with rTTCF*

Charles River (Margate, Kent, UK) supplied 7-9 week old female wild type C57Bl/6 mice and Cr2-/- hCR2+/- mice were maintained under Home Office licence 60/3868 (Dr K J Marchbank). The hCR2 transgenic mice were lambda hCR2 transgenic mice (Marchbank et al., 2002; Marchbank et al., 2000) crossed with the Cr2-/- mice developed by Dr H Molina (Molina et al., 1996) on the C57Bl/6 background. Mice were housed in conventional filter cages under pathogen-free conditions with animal husbandry provided by the staff within Comparative Biology Centre of Newcastle University (maintained under the guidelines of Newcastle University’s ethical review committee) and members of the Marchbank group. Mice were handled according to Home Office guidelines at all times and had free access to regular food and water.

*Experiments with TTCF in Denver*

These experiments were carried out in the lab of Dr L Kulik (University of Colorado Denver, CO, USA) and used the bacterial artificial chromosome (BAC)-derived hCR2 transgenic mice (Kulik et al., 2011) interbred with the Cr2-/- mice developed by Dr H Molina (Molina et al., 1996). Specifically, Cr2-/-, Cr2-/- hCR2+/+, Cr2+/+ hCR2+/+ (all on the C57Bl/6 background) and wild type C57Bl/6 mice were used and all animals were maintained under the guidelines of University of Colorado.

### Preparation and Administration of HEL Protein Vaccines

Proteins for vaccination were made up in saline solution with or without CFA (Sigma, Poole, Dorset; 1:1, v/v). Groups of 5 – 6 anaesthetised mice received subcutaneous injections (100µl) of antigen (supplementary table 2), into the scruff of the neck. Booster vaccinations of 500µg/ml native hen egg lysozyme (Sigma, Poole, UK) without adjuvant were administered at day 28.

###

### Serum Sampling and Tissue Collection

Blood samples were taken prior to immunisation and weekly from day 14. Mice were held in a tube restrainer. The tips of their tails treated with ethylchloride (Dr Georg Friedrich Henning Chemische Fabrik Walldorf GmbH, Walldorf, Germany) followed by removal of the tip of the tail (< 1 mm) with a clean scalpel. Blood was collected in Microvette CB 300 blood collection tubes (Sarstedt, Leicester, UK) and left to clot at room temperature for 2 hours. Samples were stored at 4°C overnight. The clot was pelleted by centrifugation, serum was then removed and stored in a fresh container at –20°C. Experiments were terminated at day 42 (DNA) or day 35 (proteins). Mice were sacrificed humanely according to home office guidelines. A final blood sample was taken by cardiac puncture and spleens were removed. One half of the spleen was used for FACS analysis, the other half was snap frozen in isopentane on dry ice.

###

### Vectors

#### CpG^-^ MCS

Lyopholised CpG^-^ MCS (containing a multiple cloning site) was provided by Invivogen (San Diego, USA, Figure S5). The vector and the vector-specific bacterial strain GT115 were reconstituted and handled according to the manufacturer's instructions. Vector and host had been modified to prevent formation of CpG dinucleotides, using the R6Kγ origin under control of GT115's *pir* gene. A CpG-free zeocin resistance gene facilitated bacterial selection. Mammalian expression was driven by a mouse CMV enhancer and human EF1α promoter and terminated by SV40.

#### CpG^+^ HEL and CpG^+^ hC3d^S^_3_-HEL

Vectors were originally supplied by Adprotech, now Inflazyme (actual names: pVK119-01 and pVK142-01 respectively). They were derived from the original C3d-containing DNA vaccine vectors (Barrault et al., 2005) and contained a CMV promoter and a kanamycin resistance gene (supplementary figure 2). This group of vectors contained several CpG sequences, prokaryotic DNA adjuvant sequences (supplementary table 1).

### Introduction of a Multiple Cloning Site

A multiple cloning site (MCS) was designed consisting of Bts I, Not I, Xho I, HinD III, Bgl II, Eco R I, Eco RV, Xba I. 5'-phosphorylated oligonucleotides were supplied by MWG (Ebersberg, Germany) designed to create overhangs to fit into a Bts I and Bam HI-linearised vector. Equal amounts (10 pmol) of oligonucleotide were mixed, heated to 95°C and left to cool slowly to create the double stranded insert with overhangs. CpG^+^ was digested with Bts I and Bam HI and mixed with the MCS fragment, 10mM ATP, ligase and ligation buffer. The reactions were incubated at 16°C overnight and transformed by electroporation as described before.

***Preparation and Administration of DNA Vaccine Formulations***

#### Intramuscular Injections

Large-scale DNA preparations were precipitated with ethanol and resuspended in saline solution at 2mg/ml. Groups of 5–6 mice anaesthetised with isoflurane (Baxter Healthcare Ltd., Thetford, UK) received 50µl (100µg DNA) by injection into the hind leg muscle.

#### Biolistic Delivery by Gene Gun

Transdermal (t.d.) delivery was carried out using the Helios Gene Gun^™^ System (Biorad, Hemel Hampstead, UK). Biorad provided most reagents, spermidine was from Fisher (Loughborough, UK), nitrogen and helium gas were from BOC (Guildford, UK). The coating of the ammunition was carried out according to the manufacturer's instructions except the following conditions. Microparticle loading quantitiy (MLQ) was optimised to 0.25 mg/shot 1 μm gold particles. The DNA loading ratio (DLR) was 1 µg DNA per shot relating to 4 µg DNA per mg gold. Polyvinylpyrrolidone (PVP) was diluted in 100% ethanol to a final concentration of 0.15 mg/ml. DNA-coated gold in PVP ethanol was loaded into ~33 inches Gold-Coat^™^ tubing. The tubing was inserted into the tubing preparation station; ethanol was removed using a peristaltic pump. The tubing was rotated to achieve an even distribution of gold around the tubing followed by drying for 10 minutes with nitrogen. Gold-coated tube cuttings were stored at 4°C. Mice were anaesthetised for baseline blood sampling and to shave the abdominal area. Mice were allowed to awaken from anaesthesia prior to gold being fired into two non-overlapping skin sites using ultra-pure helium at 400 psi.

### Statistical Analysis

Statistical analyses were conducted using the GraphPad software. Sets of data were compared by one-way ANOVA using the Tukey post-test and t-test. Where applicable, Kruskal-Wallis Test and Dunn's Multiple Comparison Test were used. Additional statistical analysis of the results was performed in SPSS (Version 17.0, IBM, USA) with One-way Analysis of Variance (One-way ANOVA) to compare the group means. Data normality of distribution was checked using the Shapiro-Wilk’s test and homogeneity of variances were assessed by the Levene’s test. Subsequent post-hoc test to conduct multiple comparisons between three or more group means was based on Tukey’s HSD adjustment, or independent two-sample t test for comparison of two group means.

Barrault, D.V., Steward, M., Cox, V.F., Smith, R.A., and Knight, A.M. (2005). Efficient production of complement (C3d)3 fusion proteins using the baculovirus expression vector system. Journal of immunological methods *304*, 158-173.

Kulik, L., Chen, K., Huber, B.T., and Holers, V.M. (2011). Human complement receptor type 2 (CR2/CD21) transgenic mice provide an in vivo model to study immunoregulatory effects of receptor antagonists. Molecular immunology *48*, 883-894.

Marchbank, K.J., Kulik, L., Gipson, M.G., Morgan, B.P., and Holers, V.M. (2002). Expression of human complement receptor type 2 (CD21) in mice during early B cell development results in a reduction in mature B cells and hypogammaglobulinemia. Journal of immunology *169*, 3526-3535.

Marchbank, K.J., Watson, C.C., Ritsema, D.F., and Holers, V.M. (2000). Expression of human complement receptor 2 (CR2, CD21) in Cr2-/- mice restores humoral immune function. Journal of immunology *165*, 2354-2361.

Molina, H., Holers, V.M., Li, B., Fung, Y., Mariathasan, S., Goellner, J., Strauss-Schoenberger, J., Karr, R.W., and Chaplin, D.D. (1996). Markedly impaired humoral immune response in mice deficient in complement receptors 1 and 2. Proceedings of the National Academy of Sciences of the United States of America *93*, 3357-3361.
