# Supplementary material for: A novel C3d-containing oligomeric vaccine provides insight into the viability of testing human C3d-based vaccines in mice
Source: Immunobiology. 2018 Jan;223(1):125–34. doi: 10.1016/j.imbio.2017.10.002 (PMC5849677; doi:10.1016/j.imbio.2017.10.002)
Supplement: Supplementary file 4 [file mmc4.docx]

**Supplementary table 1:** Vaccine constructs studied in CpG^-^ and CpG^+^ DNA vaccines (supplementary figures 3 and 4) including their promoters.

| **Vector** | **Promoter** | **Inserted sequence** |
| --- | --- | --- |
| CpG^-^ | EF1α | MCS – no protein |
|  |  | hC3d^S^-HEL-Oct |
|  |  | HEL-Oct |
| CpG^+^ | CMV | MCS – no protein |
|  |  | HEL |
|  |  | HEL-hC3d^S^_3_ |
|  |  | hC3d^S^-HEL-Oct |
|  |  | HEL-Oct |

**Supplementary table 2:** Concentrations of antigen administered subcutaneously

| **Antigen** | **Concentration** | **Adjuvant** |
| --- | --- | --- |
| Native hen egg lysozyme | 500 µg/ml | CFA |
| HEL Fc | 250 µg/ml | CFA |
| HEL Fc | 250 µg/ml | - |
| hC3d^S^-HEL-Oct | 100 µg/ml | - |
| HEL-Oct | 100 µg/ml | - |

**Supplementary table 3:** Steady state affinity dissociation constant. K_D_ represents the strength of the interaction, Rmax is an indicator for the activity of the surface. χ^2^ is a measure of the goodness of fit. Values represent means and SEM of triplicate experiments. n.c. = not calculated by software * amount of ligand immobilised on the surface in response units

| **Ligand** | **R_L_^*^** | **calc. R_max_** | **K_D_** | **χ^2^** |
| --- | --- | --- | --- | --- |
| hC3dg-Fc | 94.7 | 55.23 ± 0.64 | 3.17 ± 0.11 nM | 1.830 |
| hC3d^S^g-Fc | 95.7 | 60.37 ± 2.18 | 2.60 ± 0.22 nM | 1.629 |
| hC3d-Fc | 96.2 | 59.20 ± 2.91 | 5.18 ± 0.50 nM | 1.677 |
| hC3d^S^-Fc | 94.3 | 35.70 ± 0.72 | 7.66 ± 0.49 nM | 2.890 |
| mC3d^S^-Fc | 94.9 | 25.30 ± 0.06 | 1.47 ± 0.47 nM | 1.527 |
| hC3d | 96.0 | 109.00 ± 1.53 | 10.63 ± 0.22 nM | 3.650 |
|  |  |  |  |  |

**Supplementary table 4:** A complete vaccination plan of these experiments. mCR2 KO are the CR1/CR2 knockout mice original developed by Molina et al, 1996. hCR2+mCR2 KO are a bacterial artificial chromosome (BAC)-derived hCR2 transgenic mice developed on the mCR2 KO background (Kulik et al., 2011). Wild type mice are C57Bl/6 from CharlesRiver.

| **Group number** | **Antigen** | **Genotype** | **Number** |
| --- | --- | --- | --- |
| 1 | hC3d-Histag-TTCF-Oct | mCR2 KO | 5 |
| 2 | hC3d-Histag-TTCF-Oct | hCR2+ mCR2 KO | 5 |
| 3 | hC3d-Histag-TTCF-Oct | hCR2+ mCR2 | 5 |
| 4 | hC3d-Histag-TTCF-Oct | Wild type | 5 |
| 5 | Histag-TTCF-Oct | mCR2 KO | 5 |
| 6 | Histag-TTCF-Oct | hCR2+ mCR2 KO | 5 |
| 7 | CFA-rTTCF | hCR2+ mCR2 | 4 |
| 8 | rTTCF | Wild type | 5 |
